# SNPs with High Linkage Disequilibrium Increase the Explained Genetic Variance and the Reliability of Genomic Predictions

**DOI:** 10.3390/ani16020337

**Published:** 2026-01-22

**Authors:** José Guadalupe Cortes-Hernández, Felipe de Jesús Ruiz-López, Francisco Peñagaricano, Hugo H. Montaldo, Adriana García-Ruiz

**Affiliations:** 1 PhD Program in Animal Health and Production Science, National Autonomous University of Mexico, Mexico City 04510, Mexico; 2 National Center for Disciplinary Research in Animal Physiology and Improvement, National Institute of Forestry, Agriculture and Livestock Research, Ajuchitlán 76280, Querétaro, Mexico; ruiz.felipe@inifap.gob.mx; 3 Department of Animal and Dairy Sciences, University of Wisconsin, Madison, WI 53706, USA; 4 Department of Genetics and Biostatistics, Faculty of Veterinary Medicine and Zootechnics, National Autonomous University of Mexico, Mexico City 04510, Mexico

**Keywords:** GWAS, Pseudo-SNP, reliability, milk yield, fat yield, protein yield, somatic cell score, predicted genomic breeding values, Holstein

## Abstract

The objective of this study was to compare the proportion of explained genetic variance (EXGV) and the reliability of genomic breeding values (GBVs) predictions for milk yield (MY), fat yield (FY), protein yield (PY) fat percentage (FP), protein percentage (PP), and somatic cell score (SCS) in Holstein cattle. Three types of genomic information were evaluated. (a) SNP-ALL: this analysis included 88,911 single nucleotide polymorphisms (SNP) from 8290 animals. (b) HAP-PSEUDOSNP: haplotypes, defined based on high linkage disequilibrium (LD, r^2^ ≥ 0.80) between SNPs, which were encoded as pseudo-SNPs, with a total of 35,552 pseudo-SNPs and 8331 animals included. (c) SNP-HAP: analysis using only individual SNPs included in the haplotypes (without recoding); for this analysis, 33,010 SNPs and 8192 individuals were retained. All analyses were conducted using the single-step genome-wide association study method implemented in the BLUPF90 software package. The results showed that the inclusion of SNPs with high LD (SNP-HAP) increases the reliability of GBVs’ predictions compared to the SNP-ALL analysis; average reliability increased between 0.05 and 0.11. Moreover, the SNP-HAP analysis resulted in a twofold increase in the EXGV for all traits, likely due to increased estimates of individual marker effects compared to the SNP-ALL analysis.

## Simple Summary

This study aimed to evaluate and compare the proportion of explained genetic variance and the reliability of genomic breeding value predictions for six traits in Holstein cattle: milk yield, fat yield, protein yield, fat percentage, protein percentage, and somatic cell score. Three types of genomic information were tested. The first approach included 88,911 single nucleotide polymorphisms from 8290 animals. The second approach used haplotypes defined by strong linkage disequilibrium (r^2^ ≥ 0.80), encoded as pseudo-SNPs, with a total of 35,552 pseudo-SNPs from 8331 animals. The third approach analyzed only the SNPs forming haplotypes, resulting in 33,010 SNPs from 8192 animals. All analyses were performed using the single-step genome-wide association study method implemented in BLUPF90. The findings revealed that using single nucleotide polymorphisms with high linkage disequilibrium improved the reliability of genomic breeding value predictions compared with the use of single nucleotide polymorphisms in general, with average increases ranging from 0.05 to 0.11. Furthermore, analysis using single nucleotide polymorphisms with high linkage disequilibrium doubled the explained genetic variance across all traits, likely due to larger estimates of individual marker effects. Overall, the study highlights the advantages of haplotype-based information for improving prediction reliability and explaining genetic variance in Holstein cattle.

## 1. Introduction

The use of SNPs has enabled the inference of haplotype structures, thereby enhancing our understanding of gene flow and population structure in both cattle and humans [1,2]. A haplotype refers to a set of contiguous alleles within a genomic region that shares a common genealogical origin. Additionally, haplotypes can be defined as groups of SNPs that are co-inherited due to high LD or based on predefined genomic windows of arbitrary length either in mega bases or in number of SNPs [2].

The use of haplotypes in genome-wide association studies (GWAS), aimed at identifying genomic regions associated with productive, reproductive, and health-related traits typically governed by polygenic effects, has demonstrated advantages in enhancing the detection power of these methods [3,4,5]. The relevance of incorporating haplotypes into GWAS lies primarily in the nature of genetic selection in livestock, which alters the frequencies of genomic blocks rather than individual loci [2,6]. Haplotypes-based approaches sometimes can increase the likelihood of detecting quantitative trait loci (QTL) of varying sizes, especially across genomic regions with heterogeneous allele frequencies. This is largely due to the high levels of LD among SNPs, which results in low or negligible recombination rates within these blocks [4,7]. When constructing haplotypes from SNP array data for GWAS, it is crucial to consider both the density of SNP arrays and the number of SNPs per haplotype. Low-density SNP arrays (<50 K) often exhibit reduced power in GWAS due to greater average physical distances between adjacent markers (>70 kb), leading to a loss of LD information [7]. Furthermore, the number of SNPs included in each haplotype must be carefully selected, as LD between markers diminishes with increasing physical distance. For instance, Salem et al. [8] reported a decline in LD measured by D’, from 0.815 at ~30 kb to 0.578 at ~500 kb, and a decrease in r^2^ from 0.283 to 0.090 over the same range.

The use of haplotypes defined as sets of SNPs in LD rather than individual SNPs, for the prediction of GBVs using statistical methods such as best linear unbiased prediction (BLUP), has shown potential to enhance the accuracy and reliability of genomic predictions in certain scenarios [9]. This improvement is also influenced by multiple factors that determine the predictive power of the model. Some of these factors are the parameters used in haplotype construction, including the number of SNPs per haplotype and the threshold LD level (commonly measured by r^2^) between adjacent SNPs [9,10]. Additionally, trait-specific factors such as heritability, the number and effect size of underlying genes or QTLs, and the polygenic nature of the trait also influence prediction accuracy [11]. Population-related factors further affect genomic prediction. These include genetic diversity, population stratification, and historical selection pressures [12].

In various livestock populations, the use of haplotypes instead of individual SNPs has led to small changes in the accuracy of GBV predictions. For instance, in Korean Hanu cattle, the evaluations of meat quality traits reported increase in prediction accuracy, ranging from 1.3 to 4.6 percentage points, when using haplotypes [9]. Similarly, studies in Simmental cattle observed improvements in GBV accuracy of up to 11.29% for carcass traits [10]. In contrast, studies in dairy cattle have reported smaller effects. For example, genomic evaluations for MY, FY, and PY showed an increase from 1 to 2% in reliability when using haplotypes of fixed length comprising five SNPs. Moreover, when haplotype length was increased to 20 SNPs, reliability slightly decreased by approximately 2% [13].

There is still no consensus on the optimal strategy for haplotype construction; however, some studies suggest that defining haplotypes based on LD levels may be more effective than using a fixed number of SNPs. The fixed-length approach can result in spurious haplotypes formed merely from random allelic combinations [14], without considering the degree of LD between SNPs. This method overlooks the key principle of haplotype that they represent blocks of alleles with strong LD, inherited together across some generations [2,11,15]. Constructing haplotypes based on LD thresholds may better capture the underlying genomic structure and more accurately reflect biologically meaningful inheritance patterns.

On the other hand, the effects of SNPs depend on the LD between the markers and the QTLs affecting the phenotype. Levels of LD between markers vary among populations, as they are influenced by factors such as breeding objectives, selection intensity, animal adaptability, migration, mating types, and breed history [2,4].

Consequently, evaluating the use of SNPs with high LD in genomic analyses of specialized cattle breeds involves prioritizing allelic variants that occur at high frequencies within the population. These SNPs are more likely to be associated with relevant genes because of long-term artificial selection. As a result, their inclusion may increase estimated marker effects and enhance the contribution of SNPs to the EXGV of the traits of interest [2,11].

Previous studies have reported modest improvements in the reliability or accuracy of genomic predictions when using haplotypes defined by fixed SNP windows or by LD, both in dairy cattle and other species [12,13,15]. However, these approaches generally rely on recoding haplotypes as pseudo-SNPs, which increases computational complexity and implementation time [15].

In contrast, the present study advances existing research by evaluating an alternative strategy that exploits LD information without recoding haplotypes. Specifically, we assessed that the use of individual SNPs characterized by high LD (r^2^ ≥ 0.80) could increase the proportion of EXGV and the reliability of GBVs in Holstein cattle, while remaining computationally simpler and more practical for routine genomic evaluations.

The objective of this study was to evaluate three different genomic sources: (a) SNP-ALL analysis using all available individual SNPs, (b) HAP-PSEUDOSNP analysis using haplotypes encoded as pseudo-SNPs based on LD structure, and (c) SNP-HAP analysis using only individual SNPs with high LD identified during haplotypes construction, and measure their impact on the proportion of EXGV by markers, the magnitude of marker effects on GWAS, and the reliability of GBVs for MY, FY, PY, FP, PP, and SCS in a Holstein cattle population.

## 2. Materials and Methods

### 2.1. Phenotypic Information

The analysis included 640,746 records of MY corresponding to the 1st (45.26%), 2nd (33.72%), and 3rd (21.02%) lactations, previously adjusted to 305 days and mature equivalent yields from 358,857 Holstein cows born from 1979 to 2019. These records included data on 204,873 records for FY, FP, PY, and PP and 171,890 records for SCS. The SCS was expressed on a linear scale ranging from 0 to 9 (where 0 = 12.5 × 1000/mL, increasing twofold for each point, until 9 = 6400 × 1000/mL) [16]. The cows were distributed across 353 herds located in 18 states of the Mexican Republic. The pedigree file comprised 470,695 animals, including 17,220 sires and 161,757 dams, with an average pedigree depth of five generations. The data were provided by the Holstein Association of Mexico, A.C. (AHM).

### 2.2. Genomic Information

The SNPs and pseudo-SNPs used in the three analyses corresponded to 8331 genotyped animals from 7809 cows and 522 sires, with information from ~116 K SNP markers previously imputed with FindHap V2 [17]. The imputation process incorporated genotyping arrays of different densities, including BovineLD v2.0 9K (4.15%), GGP Bovine LD v3.0 26K (5.91%), GGP Super LD v4.0 26K (1.38%), BovineSNP v3 50K (9.16%), GGP LD 77K (15.42%), GGP Bovine 100K (0.03%), GGP HD 150K (59.77%), Genome-Wide BOS 1 Bovine Array 640K (2.47%), and GGP HD 777K (1.71%), ILLUMINA, San Diego, CA, USA. NEOGEN, Lansing, MI, USA and Axiom, Waltham, MA, USA [18,19,20]. The genomic data was provided by the National Center for Disciplinary Research in Animal Physiology and Improvement of the National Institute of Forestry, Agricultural and Livestock Research (CENIDFyMA-INIFAP).

In the first analysis (SNP-ALL), 8290 genotypes with 88,911 individual SNPs were included. Prior to analysis, genomic quality control was applied to exclude animals with a call rate < 0.95, or with parent–progeny conflicts; the SNPs’ filtering included the parameters of minor allele frequency (MAF) < 0.05, call rate < 0.95, or a value of *p* for the Hardy–Weinberg equilibrium test < 0.15, or monomorphic [21].

For the second analysis (HAP-PSEUDOSNP), the construction of haplotypes as pseudo-SNPs was conducted in three sequential steps. In the first one, the SNPs from the imputation process were recoded into A/B format, where 0 = BB, 1 = AB, 2 = AA, and 3, 4 and 5 were coded as 00, because this number is for missing alleles (3 = B_, 4 = A_, 5 = __). The resulting dataset was then converted to binary BED format using PLINK 1.09 software [22,23] with the --make-bed option. In the second step, haplotypes were defined using PLINK v1.07 [1,22], applying the --blocks option for block detection as described by Taliun et al. [24]. Haplo-blocks consisted of at least two SNPs and were required to meet a minimum LD threshold of r^2^ ≥ 0.80 [11]; genomic quality control parameters included the following: --maf 0.01 --mind 0.05 --geno 0.02 and --allow-no-sex. In the third step, each haplotype was recoded into pseudo-SNPs in 2, 1, and 0, corresponding to the presence of two copies, one copy, or the absence of paternal and maternal alleles, respectively, allowing only haplotypes present in more than 1% of the population [15]. This step was also performed with PLINK v1.07, resulting in a dataset of 9095 genotypes and 43,026 pseudo-SNPs. Due to the recoding of haplotypes as pseudo-SNPs, multiple haplotypes with different allele combinations can be identified within the same genomic region at the population. However, because haplotypes represent phased allele combinations across multiple SNPs, different haplotypes within the same region cannot occur simultaneously within the same chromosome segment of an individual.

Genomic quality control was applied to the pseudo-SNPs dataset, to exclude animals with a call rate < 0.95 or with parent–progeny conflicts < 1%. Additionally, pseudo-SNPs with a minor allele frequency (MAF) < 0.05, a call rate < 0.95, or a Hardy–Weinberg equilibrium *p*-value < 0.15 were removed. After filtering, the final dataset included 8331 genotypes and 35,552 pseudo-SNPs, representing the constructed haplotype. This quality control was performed using the PreGSf90 1.22 program [21].

For the third analysis (SNP-HAP), a total of the 33,010 individual SNPs contained in the haplotypes (with an LD value of r^2^ ≥ 0.80) from 8182 animals were included. Both SNP and animal genotypes were subjected to the same quality control procedures as previously described to explain what the levels of EXGV would be for the evaluated traits if only high-LD SNPs were used. Notably, these SNPs were the same as those used in the construction of haplotypes in the second analysis.

After the construction of the genomic datasets for each analysis, the estimation of marker effects (SNPs or haplotypes) and their associations with the traits of interest was performed using the single-step genome-wide association study (ssGWAS) methodology. This approach is based on the prediction of genomic breeding values (GBVs) using the single-step genomic best linear unbiased prediction (ssGBLUP) method [25,26].

### 2.3. Prediction of Genomic Breeding Values with Haplotypes or SNPs

The GBV of the six traits in the three analyses were predicted with the single-step genomic BLUP method (ssGBLUP [27,28]), using BLUPF90 2.57 software [26] for each trait and dataset. The model to obtain the GBV was

(1) $$y_{ijklmn}=\mu +\;{HYS}_{i}+{\;AGE}_{j}+{\;PE}_{k}+{\;SH}_{l}+{ANI}_{m}+e_{ijklmn}$$

where $y_{ijklmn}$ is each one of the six traits (MY, FY, PY, FP, PP, and SCS). The model’s fixed effects are as follows: μ is the general mean for each trait, ${HYS}_{i}$ is the i-th level of herd year season (6831 levels; 40 years and 2 seasons: January to June and July to December), and ${AGE}_{j}$ is the j-th age level at calving in months in each lactation classified from 1 to 9 according to its distribution: for the first lactation: 1 ≤ 23.4, 2 ≥ 23.5 and ≤25.5 and 3 ≥ 25.6; for the second one: 4 ≤ 35.9, 5 ≥ 36 and ≤39.5 and 6 ≥ 39.6; and for the third one: 7 ≤ 48.7, 8 ≥ 48.8 and ≤53.3 and 9 ≥ 53.4. The model’s random effects included ${PE}_{k}$ is the k-th level of the permanent environment; ${SH}_{l}$ is the l-th level of the sire–herd interaction (40,742 levels), ${ANI}_{m}$ is the animal effect, and $e_{ijklmn}$ is the residual effect.

### 2.4. Association Analyses for Haplotypes and SNPs

After the prediction of GBVs, the GWAS were carry out with postGSf90 1.70 program to estimate the haplotypes or SNP effects for each trait and genomic data source [21,27] and to compare whether haplotypes capture the same regions of significant SNPs.

The effects of haplotypes or SNPs were estimated as;

(2) $$\hat{a}=kDZ\prime G^{-1}\hat{u}$$

where $\hat{a}$ is the estimated effect to a haplotype or SNP, k=12Σpiqi, a scalar where $p_{i}$ is the A allele frequency for the i-th marker (SNP or haplotype), $q_{i}$ is the B allele frequency for the i-th marker, D is a weight diagonal matrix which represents the variance or weight of each haplotype or SNP as proposed by VanRaden [29], Z′ is the transposed matrix of haplotype or SNPs adjusted by allele frequencies, $G^{-1}$ is the inverse genomic relationship matrix, and $\hat{u}$ is the GBV for each trait [28].

The *p*-values for the haplotype or SNP effects were calculated according to Aguilar et al. [30]:

(3) $$p\text{-}{val}_{i}\;=\;2\left(1-\Phi \left(\left|\frac{{\hat{a}}_{i}}{sd\left({\hat{a}}_{i}\right)}\right|\right)\right)$$

where ${p\text{-}val}_{i}$ is the *p*-value for haplotype or SNP effects of the evaluated traits, Φ is the cumulative standard normal function, ${\hat{a}}_{i}$ is the estimated effect of the i-th marker (SNP or haplotype), and sd is the standard deviation of the effect for the i-th marker. The significant association threshold was defined with the Bonferroni adjustment [31] as follows: −log10(0.01/88,911 SNP) for SNP-ALL analysis, −log10(0.01/35,552 haplotypes) for HAP-PSEUDOSNP analysis, and −log10(0.01/33,010 SNP) for the SNP-HAP analysis.

### 2.5. Explained Genetic Variance by Haplotypes in HAP-PSEUDOSNP, SNPs in SNP-HAP and SNPs in SNP-ALL

The EXGV by one SNP or haplotype (${\mathrm{EXGV}}_{u}$) was defined as proposed Legarra et al. [32] and Abdel-Shafy et al. [33]:

(4) $${EXGV}_{u}=2p_{i}q_{i}{{\hat{a}}_{i}}^{2}$$

where $p_{i}$ is the A allele frequency for the i-th marker (SNP or haplotype), $q_{i}$ is the B allele frequency for the i-th marker, and ${\hat{a}}_{i}$ is the estimated effect of the i-th marker.

The EXGV for all SNP or haplotype was calculated, as done by Legarra et al. [32] and Lourenco et al. [28], with the following formula (components previously described in Formula (4)):

(5) $$EXGV=2\sum_{i}^{SNP/HAP}p_{i}q_{i}\;{{\hat{a}}_{i}}^{2}$$

The EXGV calculated using this method assesses the contribution of SNPs or haplotypes to the additive genetic variance. This assessment is based on the estimated marker effect and the allele frequency, under the assumptions of the Hardy–Weinberg equilibrium and an additive polygenic model [33,34].

The calculation of the reliability for the GBV of each trait in the three analyses was carried out with the BLUPF90 methodology [35] and an ANOVA was performed to detect possible differences between the different genomic source information with SAS 9.4 [36]:

(6) $${Reliability}_{i}=1-\left(\frac{{PEV}_{i}}{\sigma_{A}^{2}\left(1+F_{i}\right)}\right)$$

where

${Reliability}_{i}$ = Reliability of the GBV of the i-th animal for each trait.

${PEV}_{i}$ = Prediction error variance for the GBV of the ith animal.

$\sigma_{A}^{2}$ = Additive genetic variance.

$F_{i}$ = Inbreeding coefficient of the i-th animal.

## 3. Results

The SNP-ALL analysis was previously presented by Cortes-Hernández et al. [37], and this research addresses results to compare the differences with the other two analyses: HAP-PSEUDOSNP analysis, using haplotypes, and SNP-HAP analysis using only SNPs with high LD.

### 3.1. Descriptive Statistics of Haplotypes

**Figure 1:**
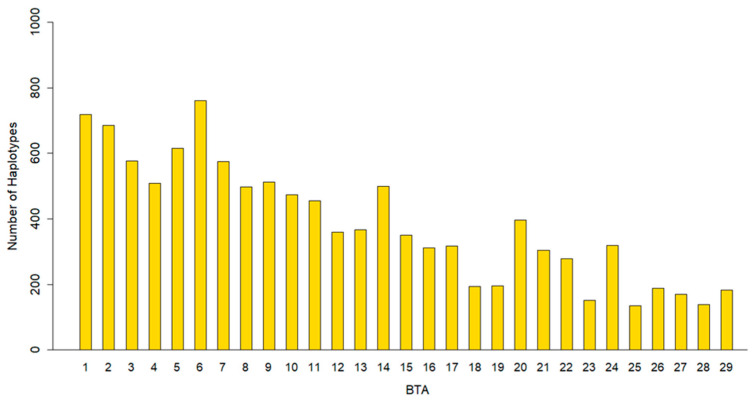
Number of haplotypes per chromosome (BTA) in Mexican Holstein cattle.

A total of 11,788 haplotypes were identified, with an average of 3.48 ± 2.44 SNPs per haplotype, ranging from a minimum of 2 to a maximum of 59 SNPs. These haplotypes resulted in the generation of 35,552 pseudo-SNPs, a largest number than the original count of haplotypes due to the presence of multiple allele combinations within the population. The average physical length of the haplotypes was 41.06 Kb, with lengths ranging from 0.003 Kb to a maximum of 199 Kb. Longer Bos taurus autosomes (BTAs) 1, 2, 3, 4, and 5 harbored a higher number of haplotypes compared to the smaller BTAs, such as BTA 26, 27, 28, and 29. Notably, BTAs 1, 2, and 6 exhibited the highest number of haplotypes. Additionally, BTAs 14, 20, and 24 showed an elevated number of haplotypes relative to their neighboring BTA (Figure 1), these results are consistent with the distribution of the pseudo-SNP by BTA.

### 3.2. Trait-Associated Haplotypes and SNPs Identified by Three Genomic Analyses

**Figure 2:**
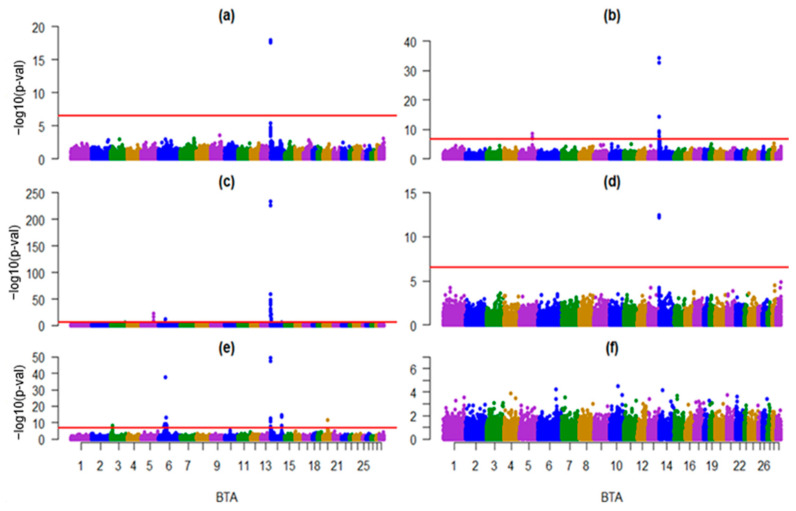
Manhattan plot for genome-wide association studies for production traits in Mexican Holstein cattle, using haplotypes (HAP-PSEUDOSNP analysis); the horizontal red line indicates the whole-genome significance threshold after Bonferroni correction at α  =  −log10(0.01/35,552). BTA: Bos taurus autosome, (**a**) milk yield, (**b**) fat yield, (**c**) fat percentage, (**d**) protein yield, (**e**) protein percentage and (**f**) somatic cell score, the different colored dots represent the *p*-values for the haplotypes on each BTA.

**Figure A1:**
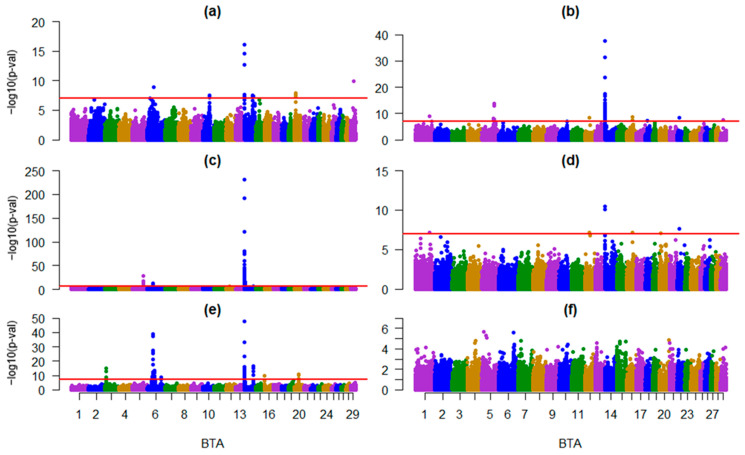
Manhattan plot for genome-wide association studies for production traits in Mexican Holstein cattle, using SNPs (SNP-ALL analysis); the horizontal red line indicates the whole-genome significance threshold after Bonferroni correction at α  =  −log10(0.01/88,911). BTA: Bos taurus autosome, (**a**) milk yield, (**b**) fat yield, (**c**) fat percentage, (**d**) protein yield, (**e**) protein percentage, and (**f**) somatic cell score, the different colored dots represent the *p*-values for the SNPs on each BTA.

**Figure A2:**
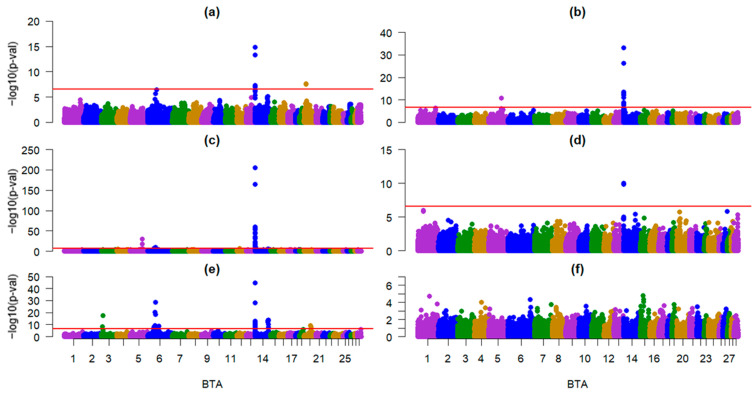
Manhattan plot for genome-wide association studies for production traits in Mexican Holstein cattle, using SNPs with high LD (r^2^ ≥ 0.80, SNP-HAP analysis); the horizontal red line indicates the whole-genome significance threshold after Bonferroni correction at α  =  −log10(0.01/33,010). BTA: Bos taurus autosome, (**a**) milk yield, (**b**) fat yield, (**c**) fat percentage, (**d**) protein yield, (**e**) protein percentage, and (**f**) somatic cell score, the different colored dots represent the *p*-values for the SNPs on each BTA.

**Figure A3:**
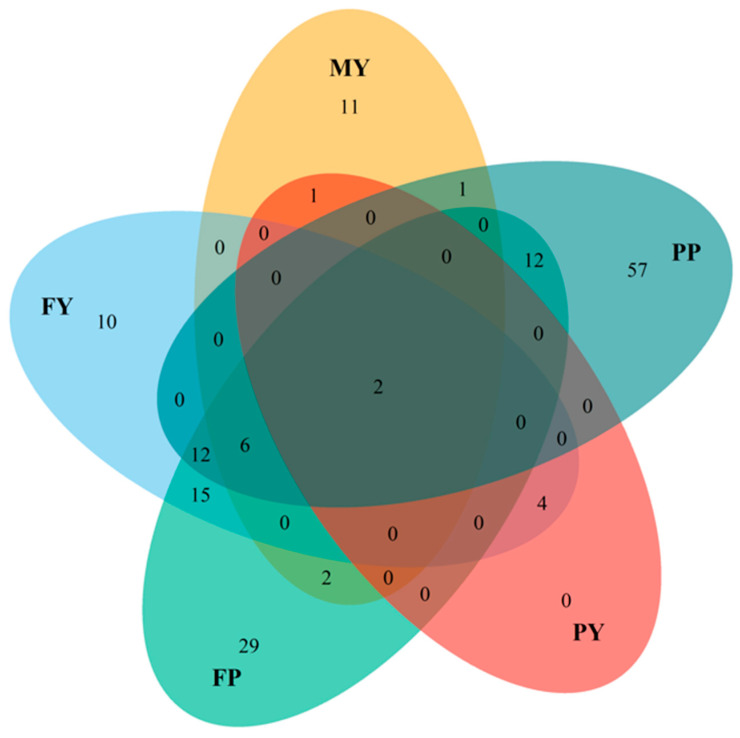
Venn diagram shows the number of SNPs uniquely associated with or shared among the traits studied in the SNP-ALL analysis: MY (milk yield), PP (protein percentage), PY (protein yield), FP (fat percentage), and FY (fat yield).

In the GWAS analyses, no haplotypes or SNPs were found to be significantly associated with SCS (Figure 2, Figure A1, Figure A2 and Figure A3). In the HAP-PSEUDOSNP analysis, 53 haplotypes were significantly associated with milk production traits, based on the significance threshold (*p* > −log10(0.01/35,552)). These associations were distributed across BTA 3, 5, 6, 14, and 20 (Figure 2; Table A1). In comparison, the SNP-HAP analysis identified 69 SNPs significantly associated with milk production traits (Figure A1), while the SNP-ALL analysis revealed 162 significant SNPs associated with the same traits (Figure A2 and Figure A3).

**Figure 3:**
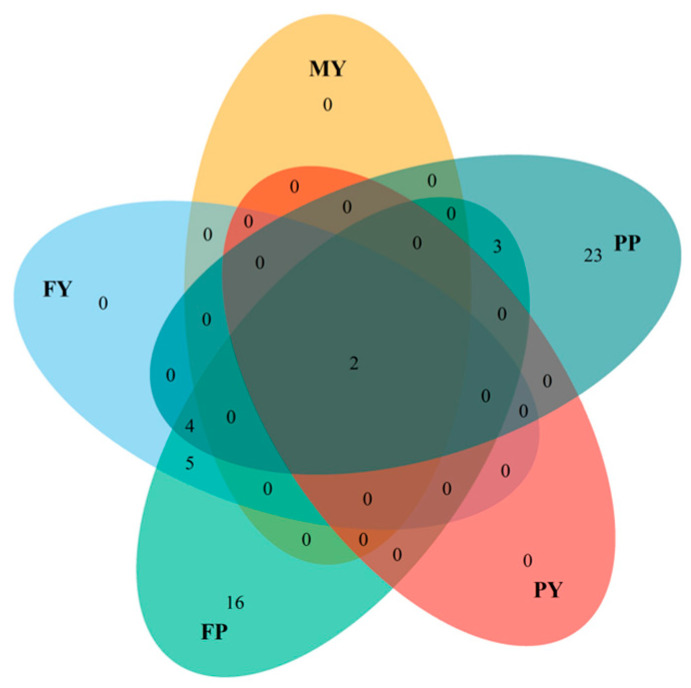
Venn diagram showing the number of haplotypes uniquely associated with or shared among the traits studied in the HAP-PSEUDOSNP analysis: MY (milk yield), PP (protein percentage), PY (protein yield), FP (fat percentage), and FY (fat yield).

From the associated haplotypes, 23 were only for PP and 16 for FP (Figure 3). Only two haplotypes (22111 and 11112, Table A1) in BTA 14 were associated with all traits (Figure 2), which were in the region of 1.51 to 1.69 Mb and 1.80 to 1.92 Mb.

In BTA 5, one haplotype was determined for two SNPs with two combinations highlighted, associated with FY and FP; in BTA 6, another haplotype of three SNPs with two combinations was associated with PP; and in BTA 20, one haplotype with nine SNPs and a single combination was associated with PP (Table A1).

**Figure 4:**
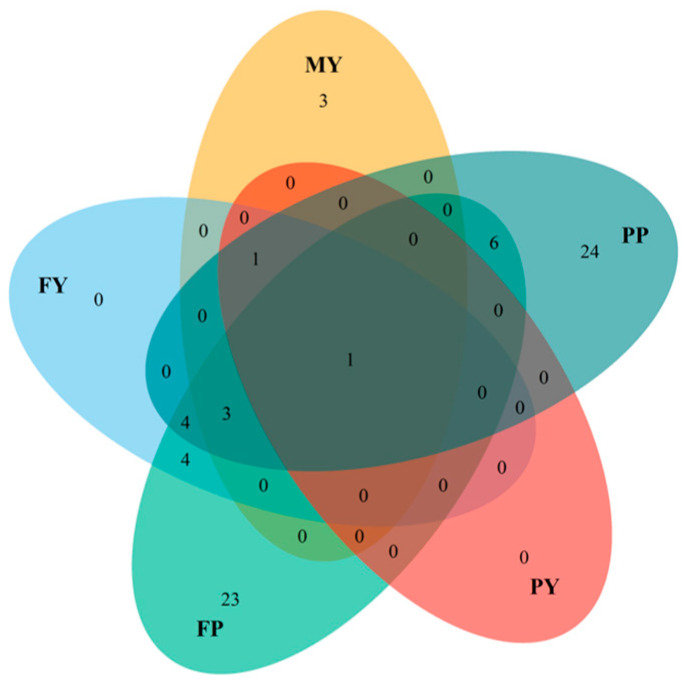
Venn diagram shows the number of SNPs uniquely associated with or shared among the traits studied in the SNP-HAP analysis: MY (milk yield), PP (protein percentage), PY (protein yield), FP (fat percentage), and FY (fat yield).

In the SNP-HAP analysis, the 69 SNPs were associated with milk production traits (Table A2) distributed in BTAs 3, 5, 6, 12, 14, and 20, with only one SNP shared among the five milk production traits. The traits that presented unique SNP associated were FP and PP, whereas FY and PY did not show unique SNP associated with (Figure 4).

### 3.3. Explained Genetic Variance and Sum of Squared Effects by SNPs and Haplotypes in the Three Analyses

A higher estimation of EXGV across all traits was observed in the SNP-HAP analysis compared to the SNP-ALL analysis (Table 1). For instance, in the case of MY, the EXGV increased from 12,636.84 ± 0.233 in the SNP-ALL analysis to 16,012.56 ± 0.904 in the HAP-PSEUDOSNP analysis and further increased to 25,798.48 ± 1.266 in the SNP-HAP analysis even though the number of markers used in the SNP-HAP analysis was lower. This suggests that the inclusion of SNPs in high LD, as identified during haplotype construction, may enhance the estimation of EXGV by better capturing the joint effects of linked loci.

Similar to the increasing rates of the EXGV for all the traits with the SNP-HAP analysis, the sum of the squared effects of the markers to MY, FY, and PY was greater in the analyses of SNP-HAP compared with the SNP-ALL analyses, but not so different between HAP-PSEUDOSNP and SNP-HAP to FP, PP, and SCS (Table 2).

### 3.4. Reliability of the GBV for the Evaluated Traits in the Three Analyses

The average reliability of GBVs increased by up to 0.11 points in the SNP-HAP analysis compared to the SNP-ALL analysis, as observed for PY. This improvement is likely due to the high LD among SNPs used in the SNP-HAP analysis, which may better capture the underlying genetic architecture of the traits. Overall, the highest reliability levels were observed for percentage traits PP and FP followed by PY and FY. In contrast, SCS exhibited the lowest reliability, ranging from 0.61 ± 0.001 to 0.64 ± 0.001 across the analyses. The reliability estimates differed significantly among the three analytical approaches (*p* < 0.0001; Table 3).

## 4. Discussion

### 4.1. Associated Haplotypes and SNPs with the Evaluated Traits

The number of haplotypes identified per BTA in this study was consistent with findings reported in other Holstein cattle populations. For example, a study conducted on 450 Holstein sires in Spain found that the longer autosomes (BTAs 1 to 5) harbored a greater number of haplotypes compared to the shorter autosomes (BTAs 26 to 29) [38]. Similarly, the average number of SNPs per haplotype observed in our study (3.48 ± 2.44) was comparable to that reported in Portuguese Holstein cattle, where the mean was 4.21 across the population. In that study, the highest number of haplotypes was also observed on BTA 1 and BTA 27 [8].

The haplotypes identified on BTAs 6, 14, and 20 that were associated with the evaluated traits correspond to previously reported quantitative trait nucleotides (QTNs). One of the most notable is the *DGAT1* gene (*Diacylglycerol O-acyltransferase 1*), located on BTA 14, which has been extensively studied and linked to MY, FY, PY, FP, and PP [39,40]. In addition, the *GHR* (*Growth Hormone Receptor*) gene on BTA 20, within the genomic region of 31.9 to 32.1 Mb, and the *ABCG2* gene, located between 37.63 and 38.41 Mb on BTA 6, were regions where associated haplotypes and SNPs were detected. These genes have previously been reported to be linked to PP and productive lifespan [41].

A study conducted on U.S. Holstein cattle under approximately 40 years of artificial selection reported a high frequency of extended haplotype homozygosity on BTA 20, specifically within the 21 to 49 Mb region, because of intense genetic selection [42]. These findings align with those observed in the present study, likely due to the historical importation of U.S. genetic material into the Mexican Holstein population. Within this same genomic region (21–49 Mb), a haplotype spanning nine SNPs and located between 31.91 and 32.10 Mb was identified in the current population and found to be significantly associated with PP (Table A1). In contrast, Holstein cattle populations from China, which do not have a direct genetic link to the animals evaluated in this study, also exhibited associations between milk production traits and nine haplotypes located in the same region of BTA 20 [43]. This convergence of evidence suggests that this genomic region harbors haplotypes that are consistently associated with productive traits, likely due to the presence of key candidate genes involved in trait expression, such as *SLC1A3*, *GHR*, *CCNB1*, and *NIPBL* [40,43].

On BTA 3, at the 15.36 Mb region, two haplotype combinations (212222222 and 222212222) were identified, composed of a set of nine SNPs and associated with PP (Table A1). This genomic region has previously been reported to harbor over 20 QTLs associated with MY, PP, FY, and FP [44]. On BTA 5, within the 93.5 to 95.6 Mb region, haplotypes were associated with FY and FP. This region includes the *ITPR2* gene, which has been linked to both MY and FY in earlier studies [45].

Although a high frequency of haplotypes was detected on BTA 24, none were significantly associated with the evaluated traits in this study. However, in Dutch Holstein cattle, loci on BTA 24 have been linked to natural antibodies in milk, which are associated with mastitis susceptibility, productive lifespan, and postpartum uterine health [46]. Additionally, a study conducted in Florida, USA, on Holstein cows reported loci in this same region related to pregnancy loss at 42 days in first- and second-lactation animals [47]. These findings may help explain the high haplotype frequency observed on BTA 24 despite the absence of trait associations in the current study.

Although no significant SNP or haplotype associations were detected for somatic cell score (SCS) across the evaluated analyses, this outcome is consistent with the highly polygenic architecture and relatively low heritability of SCS, which reduce the statistical power to detect individual marker or haplotype effects [33]. Therefore, the absence of significant associations for SCS should not be interpreted as a lack of genetic control, but rather as a limitation of marker-based association approaches for this trait.

Some haplotype blocks included a large number of SNPs (up to 59), with 12 haplotype combinations and population frequencies ranging from 1.3% to 21.8%. While such extended haplotypes may reflect genomic regions under strong historical selection, they may also increase the risk of overfitting by capturing multiple functional variants or genes with heterogeneous effects [48]. Consequently, the effects of long haplotypes should be interpreted with caution, particularly when their population frequency is low.

### 4.2. Explained Genetic Variance by SNPs, Haplotypes, and SNPs with High LD

The higher level of EXGV observed in the SNP-HAP analysis is likely attributable to the inclusion of SNPs with high LD (r^2^ > 0.80), which can enhance the detection of local additive effects among adjacent SNP [13]. These additive effects between closely linked markers have been shown to influence the overall EXGV estimation [49], supporting the findings obtained in the HAP-PSEUDOSNP analysis, where haplotypes associated with traits showed substantial explanatory power. For instance, a haplotype consisting of five SNPs located on BTA 14 (ARS-BFGL-NGS-4939, BovineHD1400000243, BovineHD1400000246, BovineHD1400000249, and Hapmap52798-ss46526455; Table A1) produced three combinations (22221, 11112, and 21112) with a population frequency greater than 1%. Among these, only the 11112 combination was significantly associated with all five evaluated traits. Notably, the individual SNPs comprising this haplotype were also independently associated with the same traits in both the SNP-ALL and SNP-HAP analyses (Table A2 and Table A3), reinforcing the importance of local LD and the additive interaction of tightly linked markers in explaining trait variation.

Unlike studies using genomic windows defined by a fixed number of SNPs [12,13], in which markers grouped within the same genomic interval may not necessarily be in LD or jointly influenced by the same underlying QTL, the use of LD-defined haplotypes may influence estimates of EXGV. Specifically, SNPs within high-LD blocks share correlated effects and are more likely to tag the same QTL [45], thereby increasing the contribution of selected markers to EXGV. As a result, EXGV values derived from LD-based groupings may be higher than those obtained using fixed-window approaches that include SNPs with weak or no LD relationships.

The estimation of EXGV was higher when using SNPs with high LD (SNP-HAP) and haplotypes coded as pseudo-SNPs (HAP-PSEUDOSNP) compared to the SNP-ALL analysis across most traits (Table 1). These results contrast with those reported by Abdel-Shafy et al. [33], who conducted a GWAS for SCS using daughter yield deviations (DYD) from German Holstein sires. In that study, the EXGV per significant SNP was approximately 2% higher than that obtained with significant haplotypes. The discrepancy with the German Holstein population may be attributed to differences in sample size (2354 genotypes), the lower number of SNPs used (44,576), and the nature of the phenotype (DYD), which may have limited the power to detect haplotype effects. Conversely, in Nelore cattle, it has been reported that haplotypes can capture epistatic interactions between variants within a haplotype locus, increasing additive genetic variance around 2% in some regions than individual SNPs for certain traits, such as meat tenderness [50]. However, the same study highlighted that longer haplotypes do not necessarily capture more genetic variance than shorter ones. This may be due to the use of fixed window sizes during haplotype construction, which does not account for regions of high LD. As a result, important marker interactions could be missed, leading to a reduced ability to detect genomic effects.

The magnitude of the EXGV observed for each trait was proportional to the magnitude of the phenotypic values. The gain in EXGV in the HAP-PSEUDOSNP analysis can be attributed to the fact that haplotype-based approaches profit larger marker effect estimates compared to individual SNPs (Table 2). This is primarily because haplotypes consist of multiple allelic combinations at a locus, increasing the likelihood that at least one of them is in linkage with a QTL. Moreover, haplotype-based models reduce the number of estimated effects while retaining relevant genomic information, effectively excluding rare alleles or those not associated with any QTL [4,51]. The greatest gains in EXGV were observed for MY, FY, and PY when using the SNP-HAP analysis. These improvements are likely due to the higher estimated effects of SNPs included in the SNP-HAP approach, which selectively incorporates markers in high LD and is more likely to capture the underlying genetic signal (Table 2).

### 4.3. Reliability of GBV in the Three Analyses and the Inclusion of SNPs with High LD in Genomic Prediction

The increase in the GBV reliability for FY, PY, and SCS from around 2% with the use of LD-defined haplotypes (HAP-PSEUDOSNP) compared to SNP-ALL analyses was found to be similar to that reported by other studies; for example, Cuyabano et al. [52] showed increases up to 3.1% for the reliability of GBV of PY when haplotypes were calculated with an r^2^ value for LD ≥ 0.75 in Nordic Holstein cattle, although they mention that increases in reliability of up to 3% can be achieved when a limit of r^2^ for LD ≥ 0.45 in low heritability traits such as fertility and mastitis. A factor to highlight in the Nordic population is the number of SNPs used for the construction of haplotypes, as they considered 492,057 SNPs and the methodology for estimated reliability. The greatest increases in the reliability of GBV were obtained with the SNP-HAP analyses, possibly since only SNPs with a high LD with r^2^ > 0.80 were included.

In contrast to the gains in reliability of GBV for the production traits with the use of haplotypes in Holstein cattle, in other cattle breeds, such as the Nelore, decreases were observed in the reliability of predictions in the same magnitude (1 to 2%) with the use of haplotypes for fatty acid traits of meat [3]. The authors attribute these results to the fact that the density of markers used was not enough to capture all LD between SNPs or haplotypes with some QTL region, because the total number of SNPs they used was 469,981 out of 893 genotypes.

The inclusion of SNPs with high LD in genomic prediction to increase the GBV accuracy and reliability of productive traits has been analyzed in other ways, for example, Mathew et al. [53] proposed the inclusion of a genomic relationship matrix adjusted by LD in the genomic prediction of different populations, showing positive results in increasing the reliability, and detecting higher EXGV, and therefore an increase of the heritability of the evaluated traits, mentioning that there are computational difficulties for the implementation of the process. Other studies suggest that the use of models with SNP selection, according to their stratification considering the LD level and the genetic structure of the trait, heritability level, and genes involved in the expression, would increase the percentage of EXGV [54], although these authors mention that these methods are not recommendable with medium-density (50 k) SNP arrays. In contrast to the results of this study about the increase in the reliability of GBV and EXGV for the traits evaluated in the SNP-HAP analyses, there were no computational problems and the number of markers used did not cause discrepant results between the evaluated traits.

On average, the use of SNPs with high LD (SNP-HAP) increases the EXGV twofold and the reliability of GBV up to 0.11, compared to the use of SNP-ALL. These increases were not found in analyses using haplotypes (HAP-PSEUDOSNP), but the findings are like those reported by other studies [51,52].

The highest increases in GBV reliability in the present study were observed in the SNP-HAP analysis, which selectively included only SNPs in strong LD (r^2^ > 0.80). This targeted selection likely enhanced predictive accuracy by focusing on SNPs more likely to tag relevant QTL regions.

The inclusion of high-LD SNPs in genomic prediction has also been explored through alternative approaches. Mathew et al. [53], for example, proposed incorporating an LD-adjusted genomic relationship matrix, which improved reliability and EXGV across diverse populations. While this approach enhanced trait heritability estimates, it was associated with notable computational challenges. Other research [54] has suggested that selecting SNPs based on LD stratification and the genetic aspects of traits (e.g., heritability, gene architecture) may improve EXGV. However, the authors cautioned against using such models with medium-density arrays (e.g., 50 K SNP chips), due to limitations in resolution and informativeness.

In contrast to those limitations, the present study demonstrated clear gains in both GBV reliability and EXGV using the SNP-HAP approach, without encountering computational difficulties or inconsistent results across traits even when using medium-density genotyping data, although the number of animal genotypes is not large.

Overall, the use of high-LD SNPs (SNP-HAP) resulted in approximately a twofold increase in EXGV and up to a 11% improvement in GBV reliability compared to the SNP-ALL analysis. While such gains were not fully replicated in the HAP-PSEUDOSNP analysis, the trends observed were consistent with findings reported by Cuyabano et al. [52] and Jónás et al. [51], reinforcing the potential of LD-informed marker selection strategies to enhance genomic prediction performance.

Although the observed 11% increase in GBV reliability may appear substantial, it is important to note that the set of markers used in the SNP-HAP analysis is not independent of the full SNPs dataset (SNP-ALL). Therefore, the observed improvement is likely driven by the strong LD among the selected SNPs.

Additionally, the higher EXGV estimated in the SNP-HAP analysis may be attributed to the larger estimated marker effects, which result from both the reduced number of markers and the strong LD structure among them. These factors directly influence the calculation of EXGV as defined in Formulas (4) and (5). Consequently, higher EXGV values obtained with this approach may not fully reflect the true genetic architecture of the evaluated traits, as the inclusion of a smaller subset of SNPs reduces genome coverage and may exclude relevant genomic regions contributing to trait expression.

## 5. Limitations

While the use of high-LD SNPs (SNP-HAP) can enhance genomic evaluations, this should be considered with caution because the set of markers in SNP-HAP represents a subset of LD-filtered SNPs, rather than an independent source of information; the improvements observed in GBV reliability are based on internal comparisons rather than external or cross-validation results, and the increase in EXGV could be reflecting inflation effects due to LD among SNPs. Additionally, several important limitations should be considered.

First, selection based on haplotypes may have antagonistic effects in certain genomic regions. For instance, a single haplotype that spans multiple genes or even different variants within the same gene may harbor alleles with opposing effects on the same trait. This could potentially reduce the overall genetic merit or increase the likelihood of expressing deleterious alleles [37,42,55]. As a result, caution is necessary when interpreting haplotype effects, especially in regions with complex gene architectures or pleiotropic effects.

Second, the generation of pseudo-SNPs from haplotypes is a labor-intensive process. It involves first defining haplotypes either by a fixed number of SNPs or by specific LD thresholds, and then reconfiguring both haplotype maps and chromosomal maps to incorporate these new markers into the genomic evaluation process. These additional steps increase the computational burden and may present challenges in routine applications.

Third, the use of SNPs in high LD for genomic evaluations requires constant updating of the selected SNP set. The addition of new markers or the inclusion of genotypes from newly analyzed animals can alter the LD structure, potentially impacting the effectiveness of previously selected SNPs. This ongoing need for recalibration makes the approach more complex and time-consuming [56]. And this also makes this process less predictable in populations with different levels of LD.

Fourth, the haplotype construction and pseudo-SNP encoding procedures were implemented using earlier versions of PLINK to ensure compatibility with established block-detection algorithms and downstream analytical pipelines. Although this choice does not affect the validity of the results, the use of more recent software versions (e.g., BEAGLE 5.5 [57]) for haplotype construction approaches may improve computational efficiency and reproducibility.

Lastly, although the use of haplotypes (like pseudoSNP) produced modest gains in EXGV and reliability of genomic predictions, the improvement over traditional SNP-based analyses was not always substantial. From a practical standpoint, the use of individual SNPs remains more straightforward, computationally efficient, and easier to implement in large-scale genetic evaluation programs.

## 6. Conclusions

Significant haplotypes in GWAS were found in the same regions as significant SNPs, and the presence of a greater number of haplotypes on BTAs 6, 14, and 20, compared to their neighboring BTAs, coincided with the presence of haplotypes associated with the evaluated traits; furthermore, associated haplotypes were also found in BTA 5.

The use of SNPs in high LD resulted in improved estimates of EXGV and marker effects across all six evaluated traits compared to using all individual SNPs (SNP-ALL). Moreover, this approach led to higher GBV reliability, particularly for FY and PY with reliability gains of up to 11%, suggesting that this method may offer a more efficient alternative for enhancing genomic predictions in Holstein cattle. However, it is essential to consider that LD patterns may change over generations, which could impact the consistency of this strategy in long-term selection schemes.

Although the use of haplotypes (like pseudoSNP) provided modest improvements in GBV reliability compared with the SNP-ALL analysis, these gains were limited and accompanied by increased computational and analytical complexity. Consequently, the HAP-PSEUDOSNP strategy should be regarded primarily as a methodological exploration rather than a practical alternative for routine genomic selection. In contrast, the SNP-HAP approach offers a more efficient and operationally feasible framework, as it leverages LD structure without requiring haplotype recoding, making it better suited for large-scale and routine genomic evaluation programs. However, further studies incorporating cross-validation or independent validation across different populations and traits are required to confirm the applicability of this methodology.

## Figures and Tables

**Table 1:** EXGV by SNPs or haplotypes in the three analyses: SNP-ALL, HAP-PSEUDOSNP, and SNP-HAP for the evaluated traits in Mexican Holstein cattle.

TRAIT	SNP-ALL (88,911 SNPs)	HAP-PSEUDOSNP (35,552 PSEUDO-SNPs)	SNP-HAP (33,010 SNPs)
MY	12,636 ± 0.233	16,012.56 ± 0.904	25,798.48 ± 1.266
FY	63.15 ± 0.001	119.21 ± 0.007	136.34 ± 0.008
PY	41.24 ± 0.001	75.25 ± 0.004	88.55 ± 0.004
FP	0.0056 ± 4.03 × 10^−7^	0.0123 ± 3.16 × 10^−6^	0.0127 ± 2.53 × 10^−6^
PP	0.0013 ± 3.14 × 10^−8^	0.0028 ± 2.22 × 10^−7^	0.0029 ± 8.56 × 10^−8^
SCS	0.0038 ± 7.04 × 10^−8^	0.0081 ± 3.96 × 10^−7^	0.0082 ± 4.13 × 10^−7^

MY: milk yield, FY: fat yield, FP: fat percentage, PY: protein yield, PP: protein percentage, and SCS: somatic cell score. SNP-ALL: Analysis using all SNP. HAP-PSEUDOSNP: Analysis using haplotypes. SNP-HAP: Analysis using SNPs with high LD (r^2^ ≥ 0.80).

**Table 2:** Sum of squared effects of genomic markers for productive traits and SCS in Mexican Holstein cattle, in the three analyses: SNP-ALL, HAP-PSEUDOSNP, and SNP-HAP.

Analysis	MY	FY	FP	PY	PP	SCS
SNP-ALL	30,127.41	151.34	0.013	99.08	0.0030	0.0091
HAP-PSEUDOSNP	40,962.65	310.02	0.032	196.39	0.0073	0.0210
SNP-HAP	60,329.48	319.68	0.030	208.48	0.0067	0.0191

MY: milk yield, FY: fat yield, FP: fat percentage, PY: protein yield, PP: protein percentage, and SCS: somatic cell score. SNP-ALL: Analysis using all SNP. HAP-PSEUDOSNP: Analysis using haplotypes. SNP-HAP: Analysis using only SNP with high LD (r^2^ ≥ 0.80).

**Table 3:** GBV reliability means and standard error for genotyped animals in the three analyses: SNP-ALL, HAP-PSEUDOSNP, and SNP-HAP for the evaluated traits in Mexican Holstein cattle.

	Type of Analysis		
TRAIT	SNP-ALL	HAP-PSEUDOSNP	SNP-HAP
MY	0.62 ± 0.001 ^a^	0.63 ± 0.001 ^b^	0.65 ± 0.001 ^c^
FY	0.69 ± 0.001 ^a^	0.71 ± 0.001 ^b^	0.79 ± 0.001 ^c^
PY	0.69 ± 0.001 ^a^	0.71 ± 0.001 ^b^	0.80 ± 0.001 ^c^
FP	0.82 ± 0.001 ^a^	0.83 ± 0.001 ^b^	0.87 ± 0.001 ^c^
PP	0.83 ± 0.001 ^a^	0.84 ± 0.001 ^b^	0.87 ± 0.001 ^c^
SCS	0.61 ± 0.001 ^a^	0.63 ± 0.001 ^b^	0.64 ± 0.001 ^c^

Different superscripts (^a, b, c^) in each row indicate a statistically significant difference (*p* < 0.0001) with ANOVA. MY: milk yield, FY: fat yield, FP: fat percentage, PY: protein yield, PP: protein percentage, and SCS: somatic cell score. SNP-ALL: Analysis using all SNP. HAP-PSEUDOSNP: Analysis using haplotypes. SNP-HAP: Analysis using only SNP with high LD (r^2^ ≥ 0.80).

## Data Availability

The phenotypic and genomic datasets generated and/or analyzed during the current study are available from the corresponding author upon reasonable request and for research purposes.

## Abbreviations

The following abbreviations are used in this manuscript:

BTA	*Bos taurus* autosome.
CR	Call rate.
END MB	End position in Mb.
EXGV	Explained genetic variance.
FP	Fat percentage.
FY	Fat yield.
GBVs	Genomic breeding values.
GWAS	Genome-wide association studies.
HAP-PSEUDOSNP	Analysis that includes haplotypes.
HAP TIP	Haplotype combination.
START MB	Start position in Mb.
LD	Linkage disequilibrium.
MAF	Minor allele frequency.
MY	Milk yield.
PP	Protein percentage.
PVAL FY	−log10(*p*-value) for fat yield.
PVAL FP	−log10(*p*-value) for fat percentage.
PVAL MY	−log10(*p*-value) for milk yield.
PVAL PY	−log10(*p*-value) for protein yield.
PVAL PP	−log10(*p*-value) for protein percentage.
PY	Protein yield.
QTL	Quantitative trait loci.
SCS	Somatic cell score.
SNP	Single nucleotide polymorphism (SNPs Plural).
SNP-ALL	Analysis included all individual single nucleotide polymorphisms.
SNP-HAP	Analysis using only individual SNPs with LD r^2^ > 0.80 (without recoding).
ssGBLUP	Single-step genomic best linear unbiased prediction method.
ssGWAS	Single-step genome-wide association study methodology.

## Appendix A

**Table A1:** Haplotypes (53) associated with production traits in Mexican Holstein cattle in genome-wide association studies for HAP-PSEUDOSNP analysis, combination of parental alleles in the haplotype, start and end positions of haplotypes in mega bases, −log10(*p*-values) in GWAS, and SNP names included in haplotypes.

HAP TIP	BTA	START MB	END MB	PVAL MY	PVAL FV	PVAL FP	PVAL PY	PVAL PP	SNP Names
**212222222**	3	15.36	15.51	-	-	-	-	8.12	ARS-BFGL-NGS-110227, ARS-BFGL-NGS-13586, BovineHD0300005023, ARS-BFGL-NGS-13057, BovineHD0300005051, ARS-BFGL-NGS-113318, BovineHD0300005068, Hapmap42643-BTA-69885, BovineHD0300005078.
**222212222**	3	15.36	15.51	-	-	-	-	6.76	ARS-BFGL-NGS-110227, ARS-BFGL-NGS-13586, BovineHD0300005023, ARS-BFGL-NGS-13057, BovineHD0300005051, ARS-BFGL-NGS-113318, BovineHD0300005068, Hapmap42643-BTA-69885, BovineHD0300005078.
**22**	5	93.92	93.93	-	-	9.25	-	-	ARS-BFGL-NGS-95906, BovineHD0500026655.
**21**	5	93.95	93.95	-	8.56	16.57	-	-	BovineHD0500026662, BovineHD0500026668.
**12**	5	93.95	93.95	-	6.88	23.09	-	-	BovineHD0500026662, BovineHD0500026668.
**12**	5	94.08	94.09	-	-	8.73	-	-	BovineHD0500026710, BovineHD0500026716.
**22222**	5	95.65	95.74	-	7.39	7.16	-	-	UA-IFASA-6009, BovineHD0500027185, BovineHD0500027187, BovineHD4100003968, BovineHD0500027195.
**1121**	6	34.63	34.66	-	-	-	-	7.84	BovineHD0600009650, BovineHD0600009653, BovineHD0600009659, BTA-21396-no-rs.
**222222**	6	37.45	37.48	-	-	-	-	9.22	BovineHD4100004481, BovineHD0600010402, BovineHD4100004482, BovineHD4100004484, Hapmap48882-BTA-75976, BovineHD0600010403.
**12221211111-** **11111111111**	6	37.80	37.85	-	-	-	-	9.09	BovineHD0600010452, Hapmap25417-BTC-036670, BovineHD0600010453, BovineHD0600010454, BovineHD0600010455, BovineHD0600010456, BovineHD0600010457, BovineHD0600010458, BovineHD0600010459, BovineHD0600010460, BovineHD0600010461, BovineHD0600010463, Hapmap43675-BTA-75814, BovineHD0600010464, BovineHD0600010465, BovineHD0600010466, BovineHD0600010467, BovineHD0600010468, BovineHD0600010469, BovineHD0600010470, BovineHD0600010471, BovineHD0600010472.
**11**	6	38.01	38.01	-	-	-	-	7.05	BovineHD0600010551, BovineHD0600010552.
**111**	6	38.28	38.29	-	-	-	-	9.03	BovineHD0600010605, BovineHD0600010606, Hapmap29922-BTC-033565.
**222**	6	38.28	38.29	-	-	-	-	7.55	BovineHD0600010605, BovineHD0600010606, Hapmap29922-BTC-033565.
**212121**	6	38.29	38.30	-	-	-	-	6.92	BovineHD4100004544, BovineHD4100004545, BovineHD0600010608, BovineHD4100004546, BovineHD0600010609, BovineHD4100004547.
**111111**	6	38.37	38.39	-	-	11.93	-	37.60	BovineHD0600010625, BovineHD4100004557, BovineHD0600010626, BovineHD0600010627, BovineHD4100004558, BovineHD4100004559.
**222222**	6	38.37	38.39	-	-	-	-	7.06	BovineHD0600010625, BovineHD4100004557, BovineHD0600010626, BovineHD0600010627, BovineHD4100004558, BovineHD4100004559.
**22222**	6	38.67	38.68	-	-	-	-	7.42	BovineHD0600010704, BovineHD0600010705, BovineHD0600010708, BovineHD0600010709, BovineHD0600010711.
**22222111111-12111111111-** **1111**	6	38.68	38.78	-	-	-	-	8.60	BovineHD0600010712, BTA-100891-no-rs, BovineHD0600010716, BovineHD0600010717, BovineHD0600010718, BovineHD0600010722, BovineHD0600010723, BovineHD0600010725, BovineHD4100004573, BovineHD0600010727, BovineHD0600010728, BovineHD0600010729, Hapmap43470-BTA-114677, BovineHD0600010730, BovineHD0600010731, BovineHD0600010733, BovineHD0600010734, BovineHD0600010735, BovineHD0600010736, BovineHD0600010737, BovineHD0600010739, BovineHD0600010740, MS-rs109570900, BovineHD0600010741. BovineHD0600010742, BovineHD0600010743.
**21**	6	39.73	39.74	-	-	-	-	12.97	BovineHD0600010897, BovineHD4100004672.
**11**	6	39.77	39.78	-	-	-	-	9.31	BovineHD0600010908, BovineHD0600010909.
**22**	6	39.77	39.78	-	-	-	-	9.33	BovineHD0600010908, BovineHD0600010909.
**11**	6	39.79	39.79	-	-	-	-	9.33	BovineHD4100004679, BovineHD0600010912.
**22**	6	39.79	39.79	-	-	-	-	9.27	BovineHD4100004679, BovineHD0600010912.
**111121**	6	39.91	39.93	-	-	-	-	7.93	BovineHD0600010931, BovineHD0600010932, BovineHD0600010933, BovineHD0600010934, BovineHD0600010935, BovineHD0600010936.
**21**	14	1.43	1.44	-	-	39.71	-	11.39	BovineHD1400000143, BovineHD1400000152.
**12**	14	1.43	1.44	-	-	30.17	-	7.11	BovineHD1400000143, BovineHD1400000152.
**22111**	14	1.51	1.70	18.00	34.28	34.09	12.20	47.25	BTA-34956-no-rs, BovineHD1400000187, ARS-BFGL-NGS-57820, ARS-BFGL-NGS-34135, ARS-BFGL-NGS-94706.
**22211**	14	1.51	1.70	-	-	25.63	-	-	BTA-34956-no-rs, BovineHD1400000187, ARS-BFGL-NGS-57820, ARS-BFGL-NGS-34135, ARS-BFGL-NGS-34135, ARS-BFGL-NGS-94706.
**11222**	14	1.51	1.70	-	-	27.14	-	-	BTA-34956-no-rs, BovineHD1400000187, ARS-BFGL-NGS-57820, ARS-BFGL-NGS-34135, ARS-BFGL-NGS-94706.
**22222**	14	1.51	1.70	-	-	18.16	-	-	BTA-34956-no-rs, BovineHD1400000187, ARS-BFGL-NGS-57820, ARS-BFGL-NGS-34135, ARS-BFGL-NGS-94706.
**22221**	14	1.80	1.92	-	9.37	58.77	-	12.73	ARS-BFGL-NGS-4939, BovineHD1400000243, BovineHD1400000246, BovineHD1400000249, Hapmap52798-ss46526455.
**11112**	14	1.80	1.92	17.60	32.87	26.79	12.51	49.16	ARS-BFGL-NGS-4939, BovineHD1400000243, BovineHD1400000246, BovineHD1400000249, Hapmap52798-ss46526455.
**21112**	14	1.80	1.92	-	7.83	31.71	-	7.72	ARS-BFGL-NGS-4939, BovineHD1400000243, BovineHD1400000246, BovineHD1400000249, Hapmap52798-ss46526455.
**11**	14	2.15	2.16	-	-	43.37	-	-	BovineHD1400000301, BovineHD1400000305.
**22**	14	2.15	2.16	-	8.79	48.42	-	7.13	BovineHD1400000301, BovineHD1400000305.
**1221**	14	2.28	2.40	-	14.42	39.92	-	10.75	BTA-35941-no-rs, ARS-BFGL-NGS-101653, ARS-BFGL-NGS-26520, BovineHD4100010534.
**2112**	14	2.28	2.40	-	6.58	18.97	-	-	BTA-35941-no-rs, ARS-BFGL-NGS-101653, ARS-BFGL-NGS-26520, BovineHD4100010534.
**2211**	14	2.71	2.76	-	-	21.21	-	-	BovineHD1400000434, ARS-BFGL-NGS-3122, ARS-BFGL-NGS-103064, BovineHD1400000447.
**1122**	14	2.71	2.76	-	-	13.08	-	-	BovineHD1400000434, ARS-BFGL-NGS-3122, ARS-BFGL-NGS-103064, BovineHD1400000447.
**22**	14	2.98	2.99	-	-	6.65	-	-	BovineHD1400000491, Hapmap24718-BTC-002945.
**2212222**	14	3.62	3.72	-	-	7.59	-	.	BovineHD1400000713, BovineHD1400000716, ARS-BFGL-NGS-74378, BovineHD1400000719, ARS-BFGL-NGS-117542, BovineHD1400000729, ARS-BFGL-BAC-17627.
**2122**	14	3.94	3.99	-	-	10.08	-	-	BovineHD1400000809, UA-IFASA-9288, BovineHD1400000817, Hapmap33328-BTC-064942.
**1222**	14	3.94	3.99	-	-	10.34	-	-	BovineHD1400000809, UA-IFASA-9288, BovineHD1400000817, Hapmap33328-BTC-064942.
**22211**	14	4.04	4.10	-	-	10.86	-	-	Hapmap32970-BTC-064990, Hapmap24986-BTC-065021, BovineHD1400000846, BovineHD1400000851, ARS-BFGL-NGS-22111.
**11**	14	4.46	4.47	-	6.60	10.71	-	-	BovineHD1400000999, UA-IFASA-5306.
**22**	14	4.46	4.47	-	-	12.51	-	-	BovineHD1400000999, UA-IFASA-5306.
**21**	14	4.57	4.58	-	-	10.24	-	-	BovineHD4100010636, Hapmap27703-BTC-053907.
**222**	14	4.77	4.78	-	-	7.13	-	-	Hapmap22692-BTC-068210, BovineHD1400001101, BovineHD1400001103.
**121211**	14	66.26	66.33	-	-	-	-	13.61	Hapmap39823-BTA-35254, BovineHD1400018536, BovineHD1400018541, Hapmap34587-BES7_Contig136_464, BovineHD1400018544, BovineHD1400018551.
**11111**	14	66.40	66.47	-	-	-	-	14.67	BovineHD1400018564, BovineHD1400018566, BovineHD1400018573, BovineHD1400018576, BovineHD1400018582.
**11**	14	66.48	66.49	-	-	-	-	8.29	BovineHD1400018585, UA-IFASA-7664.
**2222**	14	67.05	67.21	-	-	-	-	7.99	UA-IFASA-5830, BovineHD1400018761, ARS-BFGL-BAC-24806, BovineHD1400018792.
**111111121**	20	31.91	32.10	-	-	-	-	11.56	BovineHD2000009188, UA-IFASA-7069, BovineHD2000009204, BovineHD2000009215, ARS-BFGL-NGS-118998, BovineHD2000009226, BovineHD2000009234, ARS-BFGL-NGS-97963, BovineHD2000009251.

HAP TIP: haplotype combination, BTA: Bos taurus autosome, START MB: start position in Mb, END MB: end position in Mb, PVAL MY: −log10(*p*-value) for milk yield, PVAL FY: −log10(*p*-value) for fat yield, PVAL FP: −log10(*p*-value) for fat percentage, PVAL PY: −log10(*p*-value) for protein yield, PVAL PP: −log10(*p*-value) for protein percentage, SNPs: single nucleotide polymorphisms.

**Table A2:** SNPs (69) associated with production traits in Mexican Holstein cattle in genome-wide association studies for SNP-HAP analysis, positions of SNPs in mega bases, and −log10(*p*-values).

SNP Name	BTA	POS Mb	PVAL MY	PVAL FY	PVAL FP	PVAL PY	PVAL PP
**ARS-BFGL-NGS-13586**	3	15.38	-	-	-	-	8.35
**BovineHD0300005051**	3	15.45	-	-	-	-	17.59
**BovineHD0500026619**	5	93.79	-	-	6.62	-	-
**BovineHD0500026655**	5	93.93	-	-	17.21	-	-
**BovineHD0500026662**	5	93.95	-	10.75	29.70	-	-
**Hapmap60021-ss46526426**	5	95.46	-	-	6.84	-	-
**BovineHD4100004501**	6	37.59	-	-	-	-	7.05
**BovineHD0600010422**	6	37.63	-	-	-	-	6.60
**BovineHD0600010427**	6	37.68	-	-	-	-	7.86
**BovineHD0600010429**	6	37.68	-	-	-	-	6.91
**BovineHD0600010430**	6	37.68	-	-	-	-	7.05
**BovineHD0600010552**	6	38.01	-	-	-	-	9.05
**BTA-121739-no-rs**	6	38.06	-	-	8.39	-	20.10
**BovineHD0600010569**	6	38.08	-	-	8.61	-	20.27
**BovineHD0600010574**	6	38.11	-	-	-	-	8.42
**BovineHD0600010576**	6	38.12	-	-	-	-	9.15
**BovineHD0600010606**	6	38.29	-	-	-	-	6.57
**BovineHD4100004547**	6	38.30	-	-	-	-	6.73
**BovineHD0600010625**	6	38.37	-	-	7.60	-	18.40
**BovineHD4100004557**	6	38.38	-	-	7.46	-	18.36
**BovineHD4100004558**	6	38.39	-	-	9.91	-	28.73
**BovineHD0600010931**	6	39.91	-	-	-	-	8.37
**BovineHD0600010934**	6	39.91	-	-	-	-	8.68
**BovineHD0600010936**	6	39.93	-	-	-	-	8.70
**ARS-BFGL-BAC-15018**	12	30.36	-	-	6.87	-	-
**BovineHD1400000143**	14	1.43	-	-	18.10	-	-
**BovineHD1400000152**	14	1.44	-	-	23.09	-	-
**BTA-34956-no-rs**	14	1.51	-	-	15.25	-	-
**BovineHD1400000187**	14	1.59	-	-	11.66	-	-
**ARS-BFGL-NGS-57820**	14	1.65	13.36	26.37	64.45	9.83	28.34
**ARS-BFGL-NGS-34135**	14	1.68	-	13.39	46.76	-	9.39
**ARS-BFGL-NGS-94706**	14	1.70	-	12.72	44.00	-	8.78
**ARS-BFGL-NGS-4939**	14	1.80	14.83	33.06	-	10.00	44.86
**BovineHD1400000243**	14	1.87	7.02	7.64	53.00	-	11.41
**BovineHD1400000246**	14	1.88	7.18	8.75	60.49	-	12.63
**BovineHD1400000249**	14	1.89	7.36	8.26	55.57	-	12.93
**Hapmap52798-ss46526455**	14	1.92	-	-	35.14	-	7.18
**BovineHD1400000301**	14	2.15	-	-	35.14	-	-
**BovineHD1400000305**	14	2.16	-	-	34.65	-	-
**BTA-35941-no-rs**	14	2.28	-	11.05	32.44	-	11.12
**ARS-BFGL-NGS-101653**	14	2.32	-	8.58	18.55	-	-
**ARS-BFGL-NGS-26520**	14	2.39	-	10.98	20.67	-	-
**BovineHD4100010534**	14	2.40	-	12.13	30.74	-	10.34
**BovineHD1400000434**	14	2.71	-	-	8.57	-	-
**ARS-BFGL-NGS-3122**	14	2.72	-	-	8.83	-	-
**ARS-BFGL-NGS-103064**	14	2.75	-	-	15.22	-	-
**BovineHD1400000447**	14	2.76	-	-	14.85	-	-
**ARS-BFGL-NGS-74378**	14	3.64	-	-	7.61	-	-
**UA-IFASA-7076**	14	3.84	-	-	7.35	-	-
**BovineHD1400000809**	14	3.94	-	-	8.96	-	-
**UA-IFASA-9288**	14	3.96	-	-	8.63	-	-
**BovineHD1400000851**	14	4.09	-	-	8.13	-	-
**ARS-BFGL-NGS-22111**	14	4.10	-	6.52	7.91	-	-
**BovineHD1400000999**	14	4.46	-	-	12.08	-	-
**UA-IFASA-5306**	14	4.47	-	-	8.50	-	-
**Hapmap27703-BTC-053907**	14	4.58	-	-	8.72	-	-
**BovineHD1400001103**	14	4.78	-	-	9.06	-	-
**BovineHD1400018541**	14	66.28	-	-	-	-	13.52
**BovineHD1400018544**	14	66.30	-	-	-	-	11.96
**BovineHD1400018551**	14	66.33	-	-	-	-	13.18
**BovineHD1400018576**	14	66.44	-	-	-	-	10.35
**BovineHD1400018582**	14	66.47	-	-	-	-	13.80
**UA-IFASA-7664**	14	66.49	-	-	-	-	7.72
**Hapmap34051-BES7_Contig165_112**	20	5.04	7.53	-	-	-	-
**BovineHD2000001599**	20	5.05	7.68	-	-	-	-
**BovineHD2000001600**	20	5.05	7.62	-	-	-	-
**UA-IFASA-7069**	20	31.93	-	-	-	-	9.37
**BovineHD2000009226**	20	32.05	-	-	-	-	8.37
**BovineHD2000009307**	20	32.39	-	-	-	-	6.80

SNP: Single Nucleotide Polymorphism. BTA: Bos taurus autosome, POS Mb: Position in mega bases, PVAL MY: −log10(*p*-value) for milk yield, PVAL FY: −log10(*p*-value) for fat yield, PVAL FP: −log10(*p*-value) for fat percentage, PVAL PY: −log10(*p*-value) for protein yield, PVAL PP: −log10(*p*-value) for protein yield.

**Table A3:** SNPs (162) associated with production traits in Mexican Holstein cattle in genome-wide association studies for SNP-ALL analysis, and SNP position in mega bases and −log10(*p*-value).

SNP Name	BTA	POS Mb	PVAL MY	PVAL FY	PVAL FP	PVAL PY	PVAL PP
**BovineHD0100034107**	1	119.53	-	8.97	-	7.17	-
**ARS-BFGL-NGS-100206**	1	133.39	-	7.04	-	-	-
**ARS-BFGL-NGS-13586**	3	15.33	-	-	-	-	7.77
**BovineHD0300005051**	3	15.39	-	-	-	-	12.81
**ARS-BFGL-NGS-64215**	3	15.47	-	-	-	-	14.74
**BovineHD0300005253**	3	15.96	-	-	-	-	8.53
**BovineHD0500024736**	5	86.82	-	7.34	-	-	-
**BovineHD0500024796**	5	87.03	-	8.07	-	-	-
**BovineHD0500025193**	5	88.43	-	8.18	-	-	-
**BovineHD0500025605**	5	89.74	-	7.63	-	-	-
**BovineHD0500026249**	5	92.03	-	-	8.03	-	-
**BovineHD0500026635**	5	93.41	-	-	9.52	-	-
**BovineHD0500026655**	5	93.5	-	7.57	16.56	-	-
**BovineHD0500026662**	5	93.52	-	13.04	28.8	-	-
**BovineHD0500026682**	5	93.57	-	13.68	16.88	-	-
**BovineHD0500026737**	5	93.72	-	-	7.16	-	-
**BovineHD0500026872**	5	94.19	-	-	11.81	-	-
**BovineHD0500027282**	5	95.7	-	7.96	-	-	-
**BovineHD0600006457**	6	22.07	6.97	-	-	-	-
**BovineHD4100004496**	6	36.13	-	-	-	-	7.84
**Hapmap26264-BTC-037159**	6	36.16	-	-	-	-	8.49
**BovineHD4100004501**	6	36.17	-	-	-	-	8.2
**BovineHD0600010422**	6	36.2	-	-	-	-	7.37
**BovineHD0600010427**	6	36.25	-	-	-	-	8.99
**BovineHD0600010429**	6	36.26	-	-	-	-	7.96
**BovineHD0600010430**	6	36.26	-	-	-	-	8.06
**BovineHD0600010435**	6	36.3	-	-	-	-	9.37
**BovineHD0600010480**	6	36.44	-	-	-	-	6.99
**BovineHD0600010481**	6	36.44	-	-	-	-	7.23
**BovineHD0600010552**	6	36.58	-	-	-	-	10.28
**BovineHD0600010555**	6	36.59	-	-	11.61	-	26.66
**BTA-121739-no-rs**	6	36.64	-	-	10.57	-	25.48
**BovineHD0600010569**	6	36.66	-	-	10.88	-	26.58
**BovineHD0600010574**	6	36.68	-	-	-	-	12.34
**BovineHD0600010576**	6	36.69	-	-	-	-	10.71
**BovineHD0600010605**	6	36.86	-	-	-	-	10.63
**BovineHD0600010606**	6	36.86	-	-	-	-	10.48
**BovineHD4100004545**	6	36.86	-	-	-	-	9.74
**BovineHD4100004546**	6	36.86	-	-	-	-	9.51
**Hapmap29922-BTC-033565**	6	36.86	-	-	-	-	10.08
**BovineHD4100004547**	6	36.87	-	-	-	-	10.61
**Hapmap26259-BTC-033526**	6	36.89	-	-	-	-	7.7
**BovineHD0600010624**	6	36.94	-	-	7.21	-	17.59
**BovineHD0600010625**	6	36.94	-	-	8.31	-	21.45
**BovineHD4100004557**	6	36.94	-	-	8.08	-	21.35
**BovineHD4100004558**	6	36.96	-	-	12.19	-	37.43
**BovineHD0600010630**	6	36.97	-	-	13.35	-	38.91
**BovineHD4100004560**	6	36.97	-	-	10.44	-	27.23
**ARS-BFGL-NGS-112812**	6	37.19	-	-	-	-	7.15
**BovineHD4100004580**	6	37.42	-	-	-	-	7.56
**BovineHD4100004586**	6	37.52	8.88	-	-	-	9.21
**BovineHD4100004675**	6	38.32	-	-	-	-	7.44
**BovineHD0600010908**	6	38.33	-	-	-	-	7.85
**BovineHD0600010909**	6	38.33	-	-	-	-	7.86
**Hapmap27298-BTC-035654**	6	38.33	-	-	-	-	13.37
**BovineHD0600010912**	6	38.35	-	-	-	-	7.85
**BovineHD4100004679**	6	38.35	-	-	-	-	7.81
**BovineHD0600010922**	6	38.41	-	-	-	-	6.97
**BovineHD0600010931**	6	38.47	-	-	-	-	11.95
**BovineHD0600010932**	6	38.47	-	-	-	-	7.96
**BovineHD0600010933**	6	38.47	-	-	-	-	8.1
**BovineHD0600010934**	6	38.47	-	-	-	-	12.14
**BovineHD0600010936**	6	38.49	-	-	-	-	11.82
**MS-rs109570900**	6	38.78	-	-	-	-	7.38
**Hapmap33079-BTA-163567**	6	39.18	-	-	-	-	7.69
**Hapmap57625-rs29027071**	6	39.8	-	-	-	-	9.58
**BovineHD0600023906**	6	85.62	-	-	-	-	7.47
**BovineHD0600023926**	6	85.69	-	-	-	-	7.15
**BovineHD0600023965**	6	85.84	-	-	-	-	8.74
**BovineHD1000017198**	10	58.11	7.46	-	-	-	-
**BovineHD1000017422**	10	59.21	7.16	-	-	-	-
**ARS-BFGL-NGS-84473**	10	59.37	7.22	-	-	-	-
**Hapmap58345-rs29010310**	12	34.66	-	8.27	-	7.13	-
**BovineHD1400000143**	14	0.24	-	-	35.87	-	8.8
**BovineHD1400000152**	14	0.26	-	-	38.28	-	8.27
**Hapmap30381-BTC-005750**	14	0.28	-	12.93	38.55	-	11.88
**Hapmap30383-BTC-005848**	14	0.31	7.28	11.9	73.9	-	15.02
**BTA-34956-no-rs**	14	0.33	-	-	30.15	-	8.3
**BovineHD1400000187**	14	0.4	-	-	25.92	-	-
**ARS-BFGL-NGS-57820**	14	0.47	14.52	31.27	192.52	10.12	32.94
**ARS-BFGL-NGS-34135**	14	0.49	-	17.52	79.83	-	16.2
**ARS-BFGL-NGS-94706**	14	0.51	-	16.5	75.94	-	15.29
**ARS-BFGL-NGS-4939**	14	0.61	16.04	37.71	230.54	10.49	47.64
**BovineHD1400000243**	14	0.68	7.13	10.36	74.18	-	13.77
**BovineHD1400000246**	14	0.69	7.51	11.12	80.76	-	14.85
**BovineHD1400000249**	14	0.7	7.62	10.85	76.76	-	15.1
**Hapmap52798-ss46526455**	14	0.73	-	7.84	51.5	-	9.14
**ARS-BFGL-NGS-71749**	14	0.76	-	7.44	23.81	-	-
**BovineHD1400000262**	14	0.78	12.75	23.71	121.68	-	23.47
**UA-IFASA-6878**	14	0.81	7.45	7.35	59.82	-	8.59
**BovineHD1400000288**	14	0.89	-	13.75	35.73	-	9.16
**ARS-BFGL-NGS-18365**	14	0.92	-	8.2	32.97	-	-
**Hapmap30922-BTC-002021**	14	0.95	-	7.99	26.22	-	-
**BovineHD1400000301**	14	0.96	-	-	41.87	-	-
**BovineHD1400000305**	14	0.97	7.31	-	41.84	-	-
**UA-IFASA-8997**	14	1	7.14	-	29.03	-	-
**Hapmap25384-BTC-001997**	14	1.02	-	9.89	16.78	-	-
**Hapmap24715-BTC-001973**	14	1.04	-	9.1	14.44	-	-
**BTA-35941-no-rs**	14	1.08	-	12.78	34.12	-	10.67
**ARS-BFGL-NGS-101653**	14	1.12	-	10.11	19.3	-	-
**ARS-BFGL-NGS-26520**	14	1.18	-	12.29	25.94	-	-
**BovineHD4100010534**	14	1.2	-	13.94	31.24	-	10.19
**Hapmap30374-BTC-002159**	14	1.27	-	11.12	33.22	-	13.05
**BovineHD4100010542**	14	1.29	-	-	7.13	-	-
**Hapmap30086-BTC-002066**	14	1.32	-	15.16	45.6	-	11.04
**Hapmap30646-BTC-002054**	14	1.35	-	13.06	38.04	-	8.95
**BovineHD1400000401**	14	1.37	-	-	12.01	-	-
**BovineHD1400000420**	14	1.42	-	-	10.73	-	-
**BovineHD1400000434**	14	1.51	-	-	12.62	-	-
**ARS-BFGL-NGS-3122**	14	1.52	-	-	13.13	-	-
**BovineHD1400000447**	14	1.61	-	7.22	20.94	-	-
**ARS-BFGL-NGS-103064**	14	1.62	-	7.11	21.57	-	-
**BovineHD1400000453**	14	1.66	-	9.35	18.89	-	-
**ARS-BFGL-NGS-22866**	14	1.68	-	7.19	8.65	-	-
**BovineHD1400000476**	14	1.77	-	-	10.16	-	-
**BovineHD1400000479**	14	1.78	-	-	13.73	-	-
**Hapmap24717-BTC-002824**	14	1.8	-	-	9.67	-	-
**ARS-BFGL-NGS-59769**	14	1.85	-	-	21.91	-	-
**ARS-BFGL-NGS-85419**	14	2.09	-	-	7.7	-	-
**Hapmap36620-SCAFFOLD50018_7571**	14	2.14	-	7.85	25.33	-	7.66
**BovineHD1400000616**	14	2.22	-	9.79	29.8	-	8.23
**BovineHD1400000788**	14	2.84	-	-	9.5	-	-
**BovineHD1400000809**	14	2.91	-	-	9.35	-	-
**UA-IFASA-9288**	14	2.93	-	-	7.95	-	-
**BovineHD1400000851**	14	3.06	-	-	8.43	-	-
**ARS-BFGL-NGS-22111**	14	3.07	-	-	8.35	-	-
**UA-IFASA-7269**	14	3.1	-	-	8.51	-	-
**Hapmap26527-BTC-005059**	14	3.15	-	-	7.27	-	-
**ARS-BFGL-NGS-56327**	14	3.31	-	7.02	-	-	-
**ARS-BFGL-NGS-100480**	14	3.34	-	9.54	15.39	-	-
**BovineHD1400000977**	14	3.39	-	-	14.96	-	-
**BovineHD1400000999**	14	3.43	-	-	11.36	-	-
**UA-IFASA-5306**	14	3.44	-	-	8.15	-	-
**Hapmap27703-BTC-053907**	14	3.55	-	-	11.49	-	-
**UA-IFASA-6329**	14	4.06	-	-	11.52	-	-
**ARS-BFGL-NGS-115947**	14	4.46	-	-	12.49	-	-
**BovineHD1400016503**	14	57.35	7.49	-	-	-	-
**BovineHD1400016730**	14	58.15	7.27	-	-	-	-
**BovineHD1400018109**	14	62.77	7.39	-	-	-	-
**BovineHD1400018541**	14	64.08	-	-	-	-	16.22
**BovineHD1400018544**	14	64.1	-	-	-	-	14.89
**BovineHD1400018551**	14	64.13	-	-	-	-	16.09
**BovineHD1400018576**	14	64.25	-	-	-	-	13.01
**BovineHD1400018582**	14	64.27	-	-	-	-	16.35
**UA-IFASA-7664**	14	64.3	-	-	-	-	10.1
**BovineHD1400018987**	14	65.8	-	-	-	-	10.49
**Hapmap42977-BTA-55653**	16	1.97	-	-	-	-	9.78
**ARS-BFGL-NGS-35038**	16	48.29	-	7.44	-	-	-
**BTB-02095583**	16	48.33	-	8.66	-	7.13	-
**BovineHD1800004933**	18	15.64	-	7.15	-	-	-
**BovineHD2000001599**	20	5.14	7.53	-	-	-	-
**BovineHD2000001600**	20	5.14	7.48	-	-	-	-
**Hapmap34051-BES7_Contig165_112**	20	5.14	7.87	-	-	-	-
**BovineHD2000001732**	20	5.69	7.23	-	-	7.04	-
**UA-IFASA-7069**	20	31.91	-	-	-	-	10.71
**ARS-BFGL-NGS-118998**	20	32.01	-	-	-	-	6.98
**BovineHD2000009226**	20	32.03	-	-	-	-	10.05
**BovineHD2000009251**	20	32.08	-	-	-	-	7.84
**BovineHD2000009307**	20	32.37	-	-	-	-	7.86
**ARS-BFGL-NGS-110176**	22	16.68	-	8.29	-	7.58	-
**Hapmap54633-rs29021971**	29	24.12	-	7.66	-	-	-
**BTB-01023946**	29	34.56	9.86	-	-	-	-

SNP: SNP name, BTA: *Bos taurus* autosome, POS Mb: position in Mb, PVAL PL: −log10(*p*-value) for milk yield, PVAL GK: −log10(*p*-value) for fat yield, PVAL GP: −log10(*p*-value) for fat percentage, PVAL PK: −log10(*p*-value) for protein yield, PVAL PP: −log10(*p*-value) for protein percentage.
